# Coexistence and food sources of adult mosquitoes (Diptera: Culicidae) in a rural health center in Piura, Peru 2024

**DOI:** 10.17843/rpmesp.2024.413.13696

**Published:** 2024-09-03

**Authors:** Archi Alejandro Ruiz Polo, Leslie Diana Luis Arismendiz, Lourdes Viviana Barrera Rivera, Arturo Alvarado Aldana, Kelina Isbelia Saavedra Cornejo, Jose Pablo Juárez Vilchez

**Affiliations:** 1 Research and Training Center in Entomology - CICE, Sub Regional Health Directorate Luciano Castillo Colonna, Piura, Peru. Research and Training Center in Entomology - CICE Sub Regional Health Directorate Luciano Castillo Colonna Piura Peru; 2 Referral Laboratory, Sub Regional Health Directorate Luciano Castillo Colonna, Piura, Peru. Referral Laboratory Sub Regional Health Directorate Luciano Castillo Colonna Piura Peru; 3 Directorate of Integrated Health Interventions, Sub-Regional Health Directorate Luciano Castillo Colonna, Piura, Peru. Directorate of Integrated Health Interventions Sub-Regional Health Directorate Luciano Castillo Colonna Piura Peru

**Keywords:** Mosquitoes, Sexual Dimorphism, Cytochrome B, PCR, RFLP

## Abstract

This study aimed to determine the coexistence and food sources of adult mosquitoes (Diptera: Culicidae) in a rural health center in Piura, Peru by using a descriptive cross-sectional design. Entomological techniques were used to capture and identify mosquitoes, and molecular biotechnology techniques were used to identify food sources. A total of 793 specimens of the *Culex* and *Aedes* genera were found coexisting, 789 (99.5%) were *Culex quinquefasciatus*, 607 (76.9%) were males and 182 (23.1%) were females. Likewise, 4 (100%) corresponded to *Aedes aegypti* females. The food sources of *Aedes aegypti* were *Homo sapiens sapiens*, and *Homo sapiens sapiens* and *Canis familiaris* were the food sources of *Culex quinquefasciatus*. This study provides evidence that rural health centers could be acting as foci of arbovirosis, with the risk that people who come for different ailments could contract diseases transmitted by *C. quinquefasciatus* and *A. aegypti*.

## INTRODUCTION

Mosquitoes (Diptera: Culicidae) are the main vectors of tropical diseases, being responsible for causing millions of deaths in urban and rural environments ^1^. However, despite the attention given to them, their feeding behavior is still not fully understood ^2^, since there are species that feed on a wide range of vertebrates and in different degrees of specificity ^3^.

Some of the most representative species are *A. albimanus* (Wiedemann, 1821) transmitter of the *P. falciparum* parasite causing malaria ^4^; *C. quinquefasciatus* (Say, 1823) transmitter of Rift Valley fever virus, St. Louis encephalitis virus, West Nile virus, filarial and avian malaria parasites ^5^; and *A. aegypti* transmitter of dengue (DENV), chikungunya (CHIKV), and zika (ZIKV) viruses ^6^^,^^7^.

Culicid mosquitoes can coexist sharing food from a larval stage ^8^, however, they rarely coexist when they reach adulthood, since some species are anthropophilic, others zoophilic and few share both habits ^9^, thus allowing to infer vectorial capacity through feeding patterns and potential reservoirs from molecular markers such as the cytochrome B (CytB) gene of mitochondrial DNA (mtDNA) ^10^.

The CytB gene is a mtDNA marker widely used in the identification of higher organisms, its choice is based on its small size, its conserved organization, the mutation rate per site per year and the use of universal primers that amplify genes from a wide variety of vertebrates and invertebrates ^11^. Although there are studies in which this marker has already been used to identify mosquito food sources ^10^, it has not yet been used in Peru, particularly in vector species that coexist in rural health centers that could be acting as foci of infection. Therefore, this study aimed to determine the coexistence and food sources of adult mosquitoes (Diptera: Culicidae) in health care areas of the Querecotillo rural health center in the province of Sullana in January 2024.

**KEY MESSAGES**

Motivation for the study. Rural health facilities could be potential foci of transmission and scenarios of zoonosis during epidemic outbreaks of dengue and other arbovirosis due to the coexistence of mosquito species that feed on different vertebrates.

Main findings. *Aedes aegypti* feeds on *Homo sapiens sapiens*. *Culex quinquefasciatus* feeds on *Homo sapiens sapiens* and *Canis familiaris*. Both coexist in health care areas of the Querecotillo health center.

Implications. Molecular techniques should be integrated into vector control to understand feeding patterns in natural conditions and information on probable reservoirs.

## THE STUDIO

### Design and setting

**Figure 1:**
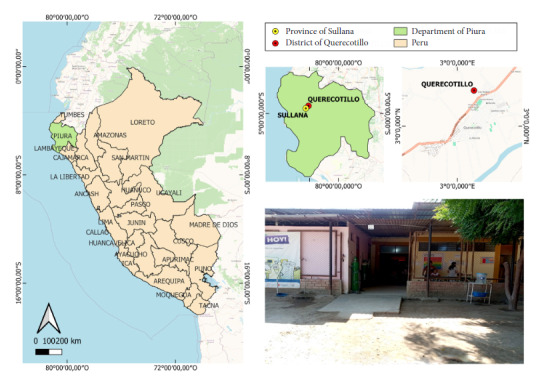
Querecotillo health center, in which adult culicid mosquitoes were collected inside healthcare areas.

We conducted a quantitative, descriptive, cross-sectional study. The study area corresponds to the Querecotillo health center, located in the rural district of Querecotillo in the province of Sullana, Peru (4° 50' 16.01“ S, 80° 38' 44.02” W) (Figure 1). This facility is characterized by having open infrastructure, and has the following areas: triage, environmental health, febrile, obstetrics hospitalization, obstetrics planning and nursing. The district had up to seven months of continuous rainfall during the El Niño phenomenon of 1993 and 1998. The weather is usually hot, and even hotter during summer, with maximum temperatures of 43.2°C and average humidity of 66%. Rainfall varies between 10 and 200 mm ^12^.

### Mosquito capture and identification

Adult mosquitoes were captured using the World Health Organization (WHO) resting capture methodology ^13^; between 4:30 p.m. (dusk) and 7:00 p.m. (night) on January 10, 12, 17, 19, 24 and 26, 2024. They were transferred to the Entomology Research and Training Center (CICE), then exposed to ethyl acetate impregnated on absorbent cotton for five minutes and taxonomically identified using the entomological guideline of the Pan American Health Organization ^14^ for *A. aegypti*, and of Consoli *et al*. ^15^ for *C. quinquefasciatus*.

### Blood collection and DNA extraction

DNA was collected and extracted by separating female mosquitoes that had visible blood residues on the abdomen from those females that did not. Then, the mosquitoes were placed on slides using a homemade protocol, then 100 uL of DNA/RNA Shield Zymo Biomics (R1100-250) preservative solution was added, and pressure was exerted with sterile toothpicks on the abdominal segment, blood was obtained and mixed with 100 uL of the solution, then aspirated and deposited in vials with 200 uL of the same solution. Blood cell DNA was extracted from the collected blood using the Zymo Biomics Kit (D4300), replacing the cell lysis step with silica microbeads with a macerate with sterile plastic pistils, and a 10,000 rpm centrifugation. Finally, we followed the factory protocol.

### PCR of the CytB gene

PCR was performed according to the indications by Chena *et al*. ^10^, and the protocol of the GoTaq™ G2 PCR kit (Promega M7801), using the primers designed by Oshagi *et al*. ^16^ (Cytb 1: 5-CCCCTCAGAATGATATATTTGTCCTCA-3 and Cytb 2: 5́-CCATCATCCAACATCTCTCAGCATGATGAAA-3). The final volume of 50 µL contained the following: 22.5μL of nuclease-free water, 10 μL of buffer (1X), 3 μL of MgCL (1.5 mM), 1 μL of dNTPs (200 μM), 2.5 μL of Forward cyt b1 (10 uM), 2.5 μL of Reverse cyt b2 (10 uM), 0.5 μL of Gotaq Polymerase enzyme (1 U/reaction) and 8 μL of DNA. Thermal conditions and cycling consisted of an initial denaturation of 95 °C for 5 min, followed by 35 cycles with 95 °C for 30 sec for denaturation, 58 °C for 30 sec for hybridization, 72 °C for 1 min for extension, a post-extension of 72 °C for 5 min and a storage temperature of 4 °C for up to 24 hours.

### Enzymatic digestion of the CytB gene and agarose gel electrophoresis

We used H*ae* III and M*wo* I enzymes that recognize RFLP in H*ae* III from *H. sapiens sapiens* and *G. gallus*; and RFLP in M*wo* I from *M. musculus* and *C. familiaris*. PCR products were digested following the factory protocols for each enzyme. Forty-five μL of PCR product, 20 μL of Buffer (1X) and 15 μL of enzyme (10 U/reaction) were mixed. H*ae* III enzyme was incubated at 37 °C for 15 min followed by 80 °C for 20 min. M*wo* I enzyme was incubated at 60 °C for 15 minutes. The reaction products were analyzed by electrophoresis on 3% agarose gels with 2.7 grams of agarose, 90 mL of 1X TAE buffer (Tris-Acetate-EDTA), 4.5 uL of ethidium bromide, 4 uL of loading dye (6X DNA loading dye) and 5 uL of sample (PCR and digestion product). The gel was exposed to 80 volts and 200 Amp for 40 minutes. The 1 kb marker (Opti-DNA Marker, G106) was used for the PCR product and the 100 bp marker (Opti-DNA Marker, G016) was used for the enzymatic digestion products.

### Data analysis

Quantitative data were tabulated in Microsoft Excel v.2021 spreadsheets and analyzed with Jamovi v.2.3.28. Molecular data were photoregistered with an Honor X7 CMA-LX3 smartphone camera and analyzed with NEBcutter™ v3.0.

### Ethical aspects

We requested consent from the physician on duty in charge of the health center prior to conducting the research, explaining the consistency and implications of the study. No patients or human samples were analyzed in this study, and therefore the approval of an institutional ethics committee was not required.

## FINDINGS

### Coexistence of mosquitoes

In the areas of the Querecotillo health center, we found a total of 793 coexisting mosquitoes between the *Culex* and *Aedes* genera; 789 (99.5%) were *C. quinquefasciatus* and only 4 (0.5%) were *A. aegypti* (Table 1). We found that 607 (76.9%) *C. quinquefasciatus* mosquitoes were male and 182 (23.1%) were female. All 4 (100%) *A. aegypti* mosquitoes were female.

**Table 1:** Number of *C. quinquefasciatus* and *A.* aegypti specimens captured in healthcare areas of the Querecotillo rural health center.

Area	Number of individuals per species		
	*A. aegypti*	*C. quinquefasciatus*	Total
Triage	0	34	34
Environmental health	2	68	70
Febrile	0	231	231
Obstetrics hospitalization	0	260	260
Obstetrical planning	2	160	162
Nursing	0	36	36
Total	4	789	793

### Mosquito feeding sources

**Figure 2:**
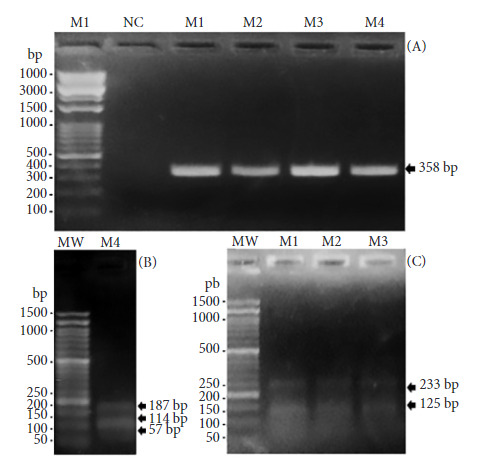
A) PCR products of the CytB gene from the blood of the abdomen of *A. aegypti.* MP: 1 kb molecular weight marker. NC: negative control. AP: *A. aegypti* pool (358 bp amplicon). P1C: pool 1 of *C. quinque-fasciatus* (358 bp amplicon). P2C: pool 2 of *C. quinquefasciatus* (358 bp amplicon). P3C: pool 3 of *C. quinquefasciatus* (358 bp amplicon). B) RFLP of the CytB gene of dog-fed *C. quinquefasciatus*. MW: 100 bp molecular weight marker. P3C: pool 3 of *C. quinquefas- ciatus* (RFLP of 187/114/57 bp). C) RFLP of the CytB gene of *A. aegypti* and *C. quinquefasciatus* fed from humans. MW: 100 bp molecular weight marker. AP: *A. aegypti pool* (233/125 bp fragments). P1C: pool 1 of *C. quinquefasciatus* (RFLP of 233/125 bp). P2C: pool 2 of *C. quinquefasciatus* (RFLP of 233/125 bp).

A total of 184 females were tested, 82 did not contain abdominal blood and 102 had recently ingested blood (2 *A. aegypti* and 100 *C. quinquefasciatus*). *A. aegypti* blood was grouped into a pool of 2 specimens (PA), and *C. quinquefasciatus* blood into two pools of 10 specimens (PC1 and PC2) and a pool of 3 specimens (PC3), discarding 77 mosquitoes due to coagulation problems during processing. We found PCR products of 358 bp and human RFLP in *A. aegypti* specimens captured in the obstetrics planning area. Human RFLP was also found in *C. quinquefasciatus* mosquitoes captured in the triage and obstetrics planning areas; and dog RFLP was found in *C. quinquefasciatus* specimens from the obstetrics hospitalization area (Table 2 and Figure 2).

**Table 2:** Food sources of *C. quinquefasciatus* and *A. aegypti* captured in healthcare areas of the Querecotillo rural health center.

Healthcare center areas	Food sources	
	*A. aegypti*	*C. quinquefasciatus*
Triage	NR	*H. sapiens sapiens* (human)
Environmental health	NR	NR
Febrile	NR	NR
Obstetrics hospitalization	NR	*C. familiaris* (dog)
Obstetrical planning	*H. sapiens sapiens* (human)	*H. sapiens sapiens* (human)
Nursing	NR	NR

NR: no record.

## DISCUSSION

In this study, we found 793 coexisting mosquitoes between *A. aegypti* (4 specimens) and *C. quinquefasciatus* (789 specimens) in the Querecotillo health center. It was not possible to analyze all the captured mosquitoes. However, in those that were analyzed, we found that *A. aegypti* fed on humans and *C. quinquefasciatus* fed on dogs and humans.

The coexistence of *A. aegypti* with *C. quinquefasciatus* and their difference in quantity in a rural area of Peru has already been reported by Ruiz *et al*. ^17^. Salazar and Moncada ^18^ reported that both species coexist in Colombia. This is explained by adaptation mechanisms by Ruiz *et al*. ^19^. The number of both species differs in most cities in tropical countries, with *C. quinquefasciatus* being approximately 20 times more abundant than *A. aegypti*^20^. Therefore, our results can be elucidated, given that we found more *C. quinquefasciatus* specimens than *A. aegypti*.

The *A. aegypti* mosquito is usually described as a species that only feeds on humans ^21^. In our results, human-feeding specimens were found in the obstetrics planning area, which is consistent with the literature. Reports from Thailand report that *A. aegypti* populations feeds on humans, cattle, pigs, cats, rats, and chickens ^22^. In the Caribbean, Fitzpatrick *et al*. ^23^ reported populations of *A. aegypti* that fed on humans, mongooses, dogs, domestic cats and wild birds.

*C. quinquefasciatus* is a mosquito with a very varied diet, which includes humans and dogs ^20^. This behavior is demonstrated by our results, since human-fed specimens were found in the triage and obstetrics planning areas. In addition, dog-fed specimens were found in the obstetrics hospitalization area. The *C. quinquefasciatus* mosquito not only feeds on humans, but also on cats, pigs, cows, horses and even reptiles ^24^. The feeding patterns of *C. quinquefasciatus* in the hemisphere exhibit highly anthropophilic behavior ^25^.

The finding of dog-feeding *C. quinquefasciatus* mosquitoes in the obstetrics hospitalization area poses a very relevant risk to the health of neonates and puerperal women receiving medical care. Previous studies have found dogs seropositive for Venezuelan equine encephalitis virus (VEEV) ^26^, which causes brain necrosis in fetuses and newborn infants when mothers are infected with VEEV during pregnancy ^27^. Likewise, some ZIKV strains have the ability to infect the *C. quinquefasciatus* mosquito ^28^, which would play a secondary role in ZIKV transmission, since *A. aegypti* is the most likely vector. However, during dengue epidemics, the flow of febrile patients to the Querecotillo health center would be a source of virus transmission. This is a relevant scenario from the epidemiological point of view, considering that *C. quinquefasciatus* that fed on dogs and humans were found coexisting with *A. aegypti* that fed on humans, since there is scientific evidence that serotypes 2 and 3 of the dengue virus have been detected in domestic dogs, which could act as potential reservoirs ^29^.

Our study has some limitations. The Querecotillo health center has an open infrastructure and is located in a rural area with a warm climate and continuous rainfall; therefore, our results only apply to this facility. In addition, the number of analyzed pools does not allow us to generalize about food sources. However, this is the first research on coexistence and food sources of arbovirus vectors within a rural health facility in Peru.

In conclusion, our results suggest that the Querecotillo health center represents a risk regarding the transmission of arbovirosis, since specimens of *A. aegypti* and *C. quinquefasciatus* were found in healthcare areas, coexisting and feeding on vertebrates other than humans, such as dogs (observed in *C. quinquefasciatus*). Research is needed to detect dengue, zika and chikungunya arboviruses in adult mosquitoes, in order to understand the transmission dynamics in rural health centers. This study contributes to the development of preventive strategies for arbovirus transmission in Sullana, Peru.
