# Supplementary figures and images for: Coexistence and food sources of adult mosquitoes (Diptera: Culicidae) in a rural health center in Piura, Peru 2024
Source: Rev Peru Med Exp Salud Publica. 2024 Sep 3;41(3):309–15. doi: 10.17843/rpmesp.2024.413.13696 (PMC11495935; doi:10.17843/rpmesp.2024.413.13696)

**Material suplementario**

**
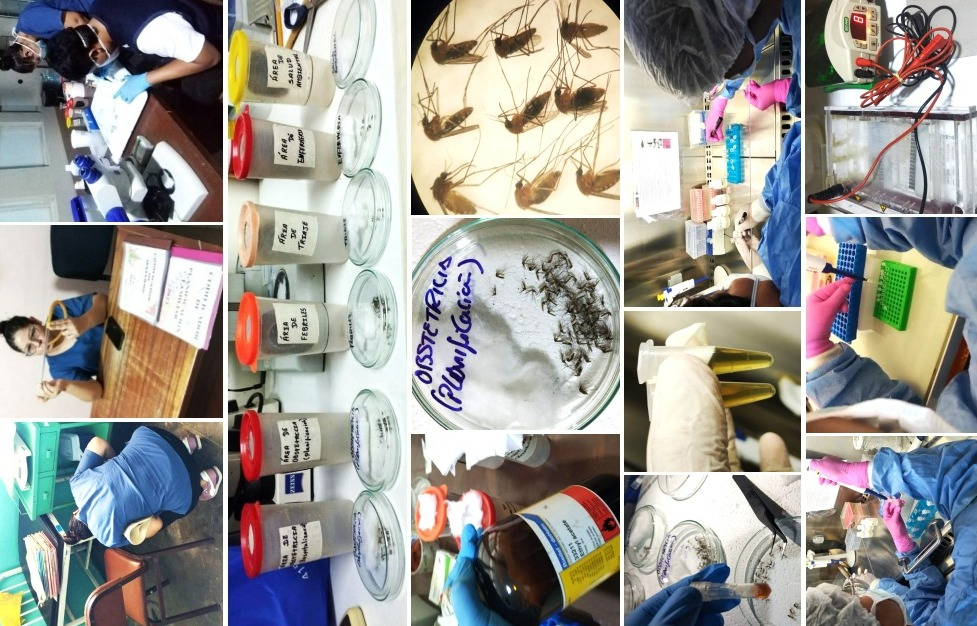
**

Supplement: Supplementary material. — Available in the electronic version of the RPMESP. [file rpmesp-41-03-13696-s001.docx]
