# Supplementary material for: Coexistence and food sources of adult mosquitoes (Diptera: Culicidae) in a rural health center in Piura, Peru 2024
Source: Rev Peru Med Exp Salud Publica. 2024 Sep 3;41(3):309–15. doi: 10.17843/rpmesp.2024.413.13696 (PMC11495935; doi:10.17843/rpmesp.2024.413.13696)
Supplement: Translation

# Translation: Coexistencia y fuentes de alimentación de mosquitos adultos (Diptera: Culicidae) en un centro de salud rural en Piura, Perú 2024

**Authors:** Archi Alejandro Ruiz Polo, Leslie Diana Luis Arismendiz, Lourdes Viviana Barrera Rivera, Arturo Alvarado Aldana, Kelina Isbelia Saavedra Cornejo, Jose Pablo Juárez Vilchez

**Affiliations:** 1 Centro de Investigación y Capacitación en Entomología - CICE, Dirección Sub Regional de Salud Luciano Castillo Colonna, Piura, Perú.; 2 Laboratorio Referencial, Dirección Sub Regional de Salud Luciano Castillo Colonna, Piura, Perú.; 3 Dirección de Intervenciones Sanitarias Integradas, Dirección Sub Regional de Salud Luciano Castillo Colonna, Piura, Perú.

**Keywords:** Mosquitos, Dimorfismo Sexual, Citocromo B, PCR, RFLP

## Abstract

Con el objetivo del estudio fue determinar la coexistencia y fuentes de alimentación de mosquitos adultos (Diptera: Culicidae) en un centro de salud rural de Piura en Perú, se realizó un estudio descriptivo transversal. Se usaron técnicas entomológicas para capturar e identificar mosquitos, y técnicas de biotecnología molecular para identificar las fuentes de alimentación. Un total de 793 ejemplares de los géneros *Culex* y *Aedes* se encontraron coexistiendo, 789 (99,5%) corresponden a *Culex quinquefasciatus,* 607 (76,9%) fueron machos y 182 (23,1%) hembras. Así mismo, 4 (100%) corresponden a *Aedes aegypti* hembras. Las fuentes de alimentación de *Aedes aegypti* fueron *Homo sapiens sapiens*, y de *Culex quinquefasciatus* fueron *Homo sapiens sapiens y Canis familiaris*. Este estudio proporciona evidencia de que los centros de salud rurales estarían actuando como focos de arbovirosis, existiendo el riesgo de que personas que acuden por distintas dolencias, puedan contraer enfermedades transmitidas por *C. quinquefasciatus* y *A. aegypti.*

## INTRODUCCIÓN

Los mosquitos (Diptera: Culicidae) son los principales vectores de enfermedades tropicales, siendo responsables de causar millones de muertes en entornos urbanos y rurales ^1^. Sin embargo, a pesar de la atención que se les brinda, aún no se termina de comprender su comportamiento alimenticio ^2^, dado que existen especies que se alimentan de una amplia gama de vertebrados y en diferentes grados de especificidad ^3^.

Entre las especies más representativas se encuentra *A. albimanus* (Wiedemann, 1821) transmisor del parásito *P. falciparum* causante de la malaria ^4^; *C. quinquefasciatus* (Say, 1823) transmisor del virus de la fiebre del Valle del Rift, el virus de la encefalitis de San Luis, el virus del Nilo Occidental, las filarias y los parásitos de la malaria aviar ^5^; y *A. aegypti* transmisor del virus del dengue (DENV), chikungunya (CHIKV), y zika (ZIKV) ^6^^,^^7^.

Los mosquitos culicidos pueden coexistir compartiendo alimento desde un estado larvario ^8^, sin embargo, al alcanzar la adultez rara vez coexisten, ya que algunas especies son antropofílicas, otras zoofílicas y pocas comparten los dos hábitos ^9^, permitiendo inferir la capacidad vectorial mediante patrones de alimentación y potenciales reservorios a partir de marcadores moleculares como el gen citocromo B (CytB) del ADN mitocondrial (ADNmt) ^10^.

El gen CytB es un marcador del ADNmt muy usado en la identificación de organismos superiores, su elección se basa en el pequeño tamaño que posee, su organización conservada, la tasa de mutación por sitio por año y el uso de cebadores universales que amplifican genes de una amplia variedad de vertebrados e invertebrados ^11^. Aunque ya existen estudios en los que se ha empleado dicho marcador para identificar fuentes alimenticias de mosquitos ^10^, aún no se ha utilizado en Perú, más aún en especies vectores que coexisten en centros de salud rurales que podrían estar actuando como focos de infección. Por tanto, el objetivo de este estudio fue determinar la coexistencia y fuentes de alimentación de mosquitos adultos (Diptera: Culicidae) en áreas de atención médica del centro de salud rural de Querecotillo de la provincia de Sullana en enero del 2024.

**MENSAJES CLAVE**

Motivación para realizar el estudio. En brotes epidémicos de dengue y otras arbovirosis, los establecimientos de salud rurales serían potenciales focos de transmisión y escenarios de zoonosis por la coexistencia de especies de mosquitos que se alimentan de distintos vertebrados.

Principales hallazgos. *Aedes aegypti s*e alimenta de Homo sapiens sapiens. *Culex quinquefasciatus* se alimenta de *Homo sapiens sapiens* y *Canis familiaris*. Ambos coexisten en areas de atención medica del centro de salud de Querecotillo*.*

Implicancias. En el control vectorial se deben integrar técnicas moleculares, que permitan comprender los patrones de alimentación en condiciones naturales y la información sobre probables reservorios.

## EL ESTUDIO

### Diseño y escenario

Se realizó un estudio cuantitativo, descriptivo, transversal. El área de estudio corresponde al centro de salud de Querecotillo, ubicado en el distrito rural de Querecotillo de la provincia de Sullana, en Perú (4° 50’ 16.01” S, 80° 38’ 44.02” O) (Figura 1). El establecimiento se caracteriza por una infraestructura abierta hacia los exteriores, dentro de la cual se brinda atención médica en las áreas de triaje, salud ambiental, febriles, hospitalización de obstetricia, planificación de obstetricia y enfermería. El distrito ha tenido lluvias de hasta siete meses continuos durante el fenómeno de El Niño de los años 1993 y 1998. Posee un clima caluroso con aumento en verano, las temperaturas máximas son de 43,2 °C y la humedad media es de 66%, las precipitaciones varían entre 10 y 200 mm ^12^.

**Figura 1:** Centro de salud de Querecotillo donde se recolectaron mosquitos culícidos adultos en el interior de áreas de atención médica.

### Captura e identificación de mosquitos

Se capturaron mosquitos adultos empleando la metodología de captura en reposo de la Organización Mundial de la Salud (OMS) ^13^; entre las 4:30 p.m. (crepúsculo) y 7:00 p.m. (noche) en los días 10, 12, 17, 19, 24 y 26 de enero del 2024. Luego, se trasladaron al Centro de Investigación y Capacitación en Entomología (CICE), se expusieron a acetato de etilo impregnado en algodón por cinco minutos e identificaron taxonómicamente usando las claves entomológicas de la Organización Panamericana de la Salud ^14^ para *A. aegypti*, y de Consoli *et al.*^15^ para *C. quinquefasciatus.*

### Obtención de sangre y extracción de ADN

La obtención y extracción de ADN se realizó separando los mosquitos hembras que presentaron residuos de sangre visibles en el abdomen de aquellas hembras que no lo presentaron. Luego, usando un protocolo casero, los mosquitos se colocaron en láminas portaobjetos, se añadió 100 uL de solución conservante DNA/RNA Shield Zymo Biomics (R1100-250), y se ejerció presión con mondadientes estériles sobre el segmento abdominal, se obtuvo la sangre y se mezcló con 100 uL más de la solución, luego se aspiró y depositó en viales con 200 uL de la misma solución. Se extrajo ADN de células sanguíneas de la sangre colectada usando el Kit Zymo Biomics (D4300), reemplazando el paso de lisis celular con microperlas de sílice por un macerado con pistilos plásticos estériles, y una centrifugación de 10 000 r. p. m. Finalmente, se siguió el protocolo de fábrica.

### PCR del gen CytB

Se realizó una PCR de acuerdo a indicaciones de Chena *et al.*^10^, y el protocolo del kit GoTaq™ G2 PCR (Promega M7801), empleando los cebadores diseñados por Oshagi *et al.*^16^ (Cytb 1: 5-CCCCTCAGAATGATATTTGTCCTCA-3 y Cytb 2: 5́-CCATCCAACATCTCAGCATGATGAAA-3). Se trabajó un volumen final de 50 µL, con 22,5μL de agua libre de nucleasas, 10 μL de buffer (1X), 3 μL de MgCL (1,5 mM), 1 μL de dNTPs (200 μM), 2,5 μL de Forward cyt b1 (10 uM), 2,5 μL de Reverse cyt b2 (10 uM), 0,5 μL de enzima Gotaq Polimerasa (1 U/reacción) y 8 μL de ADN. Las condiciones térmicas y el ciclaje consistieron en una desnaturalización inicial de 95 °C por 5 minutos, seguido de 35 ciclos con 95 °C por 30 segundos para la desnaturalización, 58 °C por 30 segundos para la hibridación, 72 °C por 1 minuto para la extensión, una posextensión de 72 °C por 5 minutos y una temperatura de conservación de 4 °C por hasta 24 horas.

### Digestión enzimática del gen CytB y electroforesis en gel de agarosa

Utilizando las enzimas H*ae* III y M*wo* I que reconoce RFLP en H*ae* III de *H. sapiens sapiens* y *G. gallus*; y RFLP en M*wo* I de *M. musculus* y *C. familiaris*, los productos PCR fueron digeridos siguiendo los protocolos de fábrica de cada enzima. Se mezclaron 45 μL de producto de PCR, 20 μL de Buffer (1X) y 15 μL de enzima (10 U/reacción). La enzima H*ae* III se incubó a 37 ºC por 15 minutos seguido de 80 ºC por 20 minutos. La enzima M*wo* I se incubó a 60 ºC durante 15 minutos. Los productos de reacción se analizaron por electroforesis en geles de agarosa al 3% con 2,7 gramos de agarosa, 90 mL de tampón TAE 1X (Tris-Acetato-EDTA), 4.5 uL de bromuro de etidio, 4 uL de colorante de carga (6X DNA loading dye) y 5 uL de muestra (producto PCR y de digestión). El gel se expuso a 80 voltios y 200 Amp por 40 minutos. Para el producto PCR se usó el marcador de 1 kb (Opti-DNA Marker, G106) y para los productos de la digestión enzimática el marcador de 100 pb (Opti-DNA Marker, G016).

### Análisis de datos

Los datos cuantitativos se tabularon en hojas de cálculo de Microsoft Excel v.2021 y se analizaron con Jamovi v.2.3.28. Los datos moleculares se fotoregistraron con la cámara de un Smartphone Honor X7 CMA-LX3 y se analizaron con NEBcutter™ v3.0.

### Aspectos éticos

Previo a la investigación se solicitó el consentimiento del médico de turno encargado del centro de salud, explicando la consistencia e implicancia del estudio. En este trabajo no se analizaron pacientes o muestras humanas, por lo que no fue necesaria la aprobación de un comité de ética institucional.

## HALLAZGOS

### Coexistencia de mosquitos

En las áreas del centro de salud de Querecotillo se halló coexistiendo un total de 793 mosquitos entre los géneros *Culex* y *Aedes*; 789 (99,5%) correspondían a *C. quinquefasciatus* y solo 4 (0,5%) a *A. aegypti* (Tabla 1). En *C. quinquefasciatus*, 607 (76,9%) fueron machos y 182 (23,1%) hembras; y en *A. aegypti* 4 (100%) fueron hembras.

**Tabla 1:** Número de ejemplares *C. quinquefasciatus* y *A. aegypti* capturados en áreas de atención médica del centro de salud rural de Querecotillo.

Área	Número de individuos por especie		
	*A. aegypti*	*C. quinquefasciatus*	Total
Triaje	0	34	34
Salud ambiental	2	68	70
Febriles	0	231	231
Hospitalización de obstetricia	0	260	260
Planificación de obstetricia	2	160	162
Enfermería	0	36	36
Total	4	789	793

### Fuentes de alimentación de mosquitos

Se analizaron 184 hembras, 82 no contenían sangre abdominal y 102 habían ingerido sangre recientemente (2 *A. aegypti* y 100 *C. quinquefasciatus*). La sangre de *A. aegypti* se agrupó en un *pool* de 2 ejemplares (PA), y la sangre de *C. quinquefasciatus* en dos *pooles* de 10 ejemplares (PC1 y PC2) y un *pool* de 3 ejemplares (PC3), descartando 77 mosquitos por problemas de coagulación durante el proceso. Se observó productos PCR de 358 pb y RFLP de humano en *A. aegypti* capturados en el área de planificación de obstetricia. También se observó RFLP de humano en *C. quinquefasciatus* capturados en las áreas de triaje y de planificación de obstetricia; y RFLP de perro en *C. quinquefasciatus* en el área de hospitalización de obstetricia (Tabla 2 y Figura 2).

**Tabla 2:** Fuentes de alimentación de *C. quinquefasciatus* y *A. aegypti* capturados en áreas de atención médica del centro de salud rural de Querecotillo.

Áreas en el centro de salud	Fuentes de alimentación	
	*A. aegypti*	*C. quinquefasciatus*
Triaje	nr	*H. sapiens sapiens* (humano)
Salud ambiental	nr	nr
Febriles	nr	nr
Hospitalización de obstetricia	nr	*C. familiaris* (perro)
Planificación de obstetricia	*H. sapiens sapiens* (humano)	*H. sapiens sapiens* (humano)
Enfermería	nr	nr

nr: no registra.

**Figura 2:** A) Productos PCR del gen CytB de la sangre del abdomen de *A. aegypti*. MP: marcador de peso molecular 1 kb. CN: control negativo. PA: *pool* de *A. aegypti* (amplicón de 358 pb). P1C: pool 1 de *C. quinque-fasciatus* (amplicón de 358 pb). P2C: *pool* 2 de *C. quinquefasciatus* (amplicón de 358 pb). P3C: *pool* 3 de *C. quinquefasciatus* (amplicón de 358 pb). B) RFLP del gen CytB de C. quinquefasciatus alimentado de perro. MP: marcador de peso molecular 100 pb. P3C: *pool* 3 de *C. quinquefasciatus* (RFLP de 187/114/57 pb). C) RFLP del gen CytB de *A. aegypti* y *C. quinquefasciatus* alimentados de humanos. MP: marcador de peso molecular 100 pb. PA: *pool* de *A. aegypti* (fragmentos de 233/125 pb). P1C: *pool* 1 de *C. quinquefasciatus* (RFLP de 233/125 pb). P2C: *pool* 2 de *C. quinquefasciatus* (RFLP de 233/125 pb)

## DISCUSIÓN

En el presente estudio se halló coexistiendo 793 mosquitos entre *A.aegypti* (4 ejemplares) y *C. quinquefasciatus* (789 ejemplares) en el centro de salud de Querecotillo. No se logró analizar todos los mosquitos capturados. Sin embargo, en los que se analizaron, se encontró que *A. aegypti* se alimentó de humanos y *C. quinquefasciatus* de perros y humanos.

La coexistencia de *A. aegypti* con *C. quinquefasciatus* y su diferencia de abundancia en una zona rural del Perú ya ha sido reportada por Ruiz *et al.*^17^. Salazar y Moncada ^18^ reportaron que ambas especies coexisten en Colombia. Esto es explicado por mecanismos de adaptación de Ruiz *et al.*^19^. La cantidad de ambas especies difiere en la mayoría de ciudades de países tropicales, siendo posible encontrar a *C. quinquefasciatus* aproximadamente 20 veces más abundante que *A. aegypti*^20^. Por tanto, nuestros resultados se pueden dilucidar, dado que hallamos a *C. quinquefasciatus* con una mayor población que *A. aegypti*.

En la literatura, el mosquito *A. aegypti* se describe como una especie que solo se alimenta del ser humano ^21^. En nuestros resultados se encontraron ejemplares alimentados de humanos en el área de planificación de obstetricia, hecho que concuerda con la literatura. En Tailandia se han reportado poblaciones de *A. aegypti* alimentadas de humanos, bovinos, cerdos, gatos, ratas y pollos ^22^. En el Caribe, Fitzpatrick *et al.*^23^, reportaron poblaciones de *A. aegypti* alimentados de humanos, mangostas, perros, gatos domésticos y aves silvestres.

*C. quinquefasciatus* es un mosquito con una alimentación muy variada en la que humanos y perros forman parte de su dieta ^20^. Un comportamiento observado en nuestros resultados, ya que se halló ejemplares alimentados de humanos en las áreas de triaje y planificación de obstetricia. Además, de ejemplares alimentados de perro en el área de hospitalización de obstetricia. El mosquito *C. quinquefasciatus* no solo se alimenta de humanos, sino también de gatos, puercos, vacas, caballos e incluso reptiles ^24^. Los patrones alimenticios de *C. quinquefasciatus* en el hemisferio exhiben un comportamiento altamente antropofílico ^25^.

El haber hallado que en el área de hospitalización de obstetricia se encuentran mosquitos *C. quinquefasciatus* alimentados de perro, suponen un riesgo muy relevante para la salud de neonatos y puérperas a quienes se les brinda atención médica. Estudios previos han encontrado perros seropositivos para el virus de la encefalitis equina venezolana (VEEV) ^26^, que causa la necrosis cerebral en fetos y niños recién nacidos cuando las madres se infectan con VEVV durante la gestación ^27^. Así mismo, existen cepas ZIKV con la capacidad de infectar al mosquito *C. quinquefasciatus*^28^, quien cumpliría un rol secundario en la transmisión del ZIKV, puesto que *A. aegypti* es el vector más probable. No obstante, en epidemias de dengue, el ingreso y salida de febriles (personas con fiebre) al centro de salud de Querecotillo, ocasionaría un foco de transmisión del virus. Un escenario relevante desde el punto de vista epidemiológico considerando que se hallaron *C. quinquefasciatus* alimentados de perro y humano, coexistiendo con *A. aegypti* alimentados de humano; puesto que hay evidencia científica en la que los serotipos 2 y 3 del virus dengue se ha detectado en perros domésticos, quienes actuarían como potenciales reservorios ^29^.

En el presente estudio se deben mencionar limitaciones. Primero, el centro de salud de Querecotillo posee una infraestructura abierta hacia los exteriores y se encuentra ubicado en una zona rural con un clima cálido con continuas lluvias, por ello nuestros resultados solo se aplican a dicho establecimiento. Segundo, el número de *pooles* analizados no permite hacer generalizaciones en cuanto a las fuentes de alimentación. Sin embargo, esta es la primera investigación que estudia la coexistencia y fuentes de alimentación de vectores de arbovirus dentro de un establecimiento de salud rural en Perú.

En conclusión, se sugiere que el centro de salud de Querecotillo, representa un riesgo en la transmisión de arbovirosis, ya que se encontraron ejemplares de *A. aegypti* y *C. quinquefasciatus* en áreas de atención médica, coexistiendo y alimentándose de vertebrados diferentes al humano, como el perro (observado en C*. quinquefasciatus*). Se necesitan investigaciones en las que se detecte los arbovirus del dengue, zika y chikungunya en mosquitos adultos con el fin de conocer la dinámica de transmisión en centros de salud rurales. Este estudio contribuye al planteamiento de estrategias preventivas en la transmisión de arbovirus en Sullana, Perú.
